# Identifying Subsets of Cancer Patients with an Increased Risk of Developing Cutaneous Melanoma: A Surveillance, Epidemiology, and End Results–Based Analysis

**DOI:** 10.1016/j.xjidi.2024.100323

**Published:** 2024-11-06

**Authors:** Thomas Z. Rohan, Jenna L. Mandel, Henry Y. Yang, Lauren Banner, Daniel Joffe, Rachel Zachian, Jaanvi Mehta, Safiyyah Bhatti, Tingting Zhan, Neda Nikbakht

**Affiliations:** 1 Department of Dermatology & Cutaneous Biology, Sidney Kimmel Medical College, Thomas Jefferson University, Philadelphia, Pennsylvania, USA; 2 Department of Internal Medicine, Lankenau Medical Center, Wynnewood, Pennsylvania, USA

**Keywords:** Cancer, Melanoma, Second primary cancer, Second primary malignancy, Surveillance, Epidemiology, and End Results

## Abstract

Cancer survivors have an increased risk of developing second primary malignancies. We aimed to identify whether certain cancers lead to an increased risk of developing melanoma among cancer survivors. We evaluated the risk of developing cutaneous melanoma after the 20 most common cancers in the United States through the Surveillance, Epidemiology, and End Results database. We identified 9 primary cancers linked to increased risk of developing a subsequent cutaneous melanoma: cutaneous melanoma (standardized incidence ratio [SIR] = 9.65), leukemia (SIR = 1.76), non-Hodgkin lymphoma (SIR = 1.33), thyroid cancer (SIR = 1.32), brain and nervous system cancer (SIR = 1.31), myeloma (SIR = 1.23), breast cancer (SIR = 1.13), oral cavity/pharynx cancer (SIR= 1.12), and prostate cancer (SIR = 1.03). The risk of developing melanoma was highest 1–5 years after diagnosis of most primary cancers. Notably, individuals aged under 50 years with a prior melanoma had a 14-fold increased risk. Our findings highlight specific at-risk groups—such as those aged under 50 years with recent melanoma, individuals in their 60s diagnosed with leukemia, and those aged over 80 years with recent thyroid cancer—who may benefit from heightened clinical vigilance and tailored melanoma screening strategies.

## Introduction

In the United States, cutaneous melanoma is the fifth most common cancer, responsible for 1.3% of all cancer deaths (American Cancer Society, 2023). Early detection and screening for melanoma, especially in at-risk groups, may reduce future mortality and morbidity (Johansson et al, 2019). Cancer survivors are at higher risk for developing subsequent cancers separate from the original malignancy, defined as second primary malignancies (Demoor-Goldschmidt and de Vathaire, 2019). Melanoma is a commonly identified second primary malignancy after some cancers, but to date, there has not been a cumulative study summarizing melanoma risk after the most common cancers (Morton et al, 2014; Siegel et al, 2023; Ugai et al, 2022). Characterization of malignancies associated with a higher risk of developing subsequent melanoma can identify patient populations that may benefit from targeted skin cancer screening. We aimed to identify which cancer types are associated with an increased risk of developing cutaneous melanoma among survivors of the 20 most common cancers in the United States. In addition, we evaluated the risk of melanoma in these cancer survivors by age at diagnosis of the first cancer, latency from initial diagnosis, and sex.

## Results

We analyzed data from the National Cancer Institute’s Surveillance, Epidemiology, and End Results, a U.S. registry of more than 9 million cancer cases (Ugai et al, 2022). We queried the Surveillance, Epidemiology, and End Results 17 database to include all primary cancers diagnosed between January 1, 2000, and December 31, 2020. The highest risk of melanoma as a second primary malignancy was in patients whose first primary cancer was also melanoma (standardized incidence ratio [SIR] = 9.65; 95% confidence interval [CI] = 9.5–9.79). The 8 other cancers with an increased risk of subsequent melanoma, ranked by their associated relative risk, were leukemia (SIR = 1.76; 95% CI = 1.65–1.86), non-Hodgkin lymphoma (SIR = 1.33; 95% CI = 1.26–1.40), thyroid cancer (SIR = 1.32; 95% CI = 1.23–1.41), brain and nervous system cancer (SIR = 1.31; 95% CI = 1.09–1.57), myeloma (SIR = 1.23, 95% CI = 1.09–1.37), breast cancer (SIR = 1.13, 95% CI = 1.09–1.17), oral cavity/pharynx cancer (SIR = 1.12; 95% CI = 1.04–1.21), and prostate cancer (SIR = 1.03; 95% CI = 1.01–1.05) (Table 1). Of these, the risk of melanoma was increased regardless of sex, except for breast (females only) and prostate (males only) cancers. Brain cancers also had a significant risk of future melanoma in males only (SIR = 1.33; 95% CI = 1.06–1.66).

**Table 1:** Cancers Associated With a Significantly Increased Risk of Subsequent Melanoma

Site of Primary Cancer (n)	Total			Male			Female		
	SIR (95% CI)	Unadjusted *P*-Value	Adjusted *P-*Value	SIR (95% CI)	Unadjusted *P-*Value	Adjusted *P-*Value	SIR (95% CI)	Unadjusted *P-*Value	Adjusted *P-*Value
Cutaneous melanoma (283,345)	9.65 (9.5–9.79)^1^	<.01	<.01	8.86 (8.7–9.02)^1^	<.01	<.01	11.86 (11.54–12.17)^1^	<.01	<.01
Leukemia (166,606)	1.76 (1.65–1.86)^1^	<.01	<.01	1.79 (1.67–1.91)^1^	<.01	<.01	1.67 (1.47–1.89)^1^	<.01	<.01
NHL (251,700)	1.33 (1.26–1.40)^1^	<.01	<.01	1.30 (1.22–1.39)^1^	<.01	<.01	1.39 (1.27–1.52)^1^	<.01	<.01
Thyroid (181,983)	1.32 (1.23–1.41)^1^	<.01	<.01	1.39 (1.24–1.56)^1^	<.01	<.01	1.27 (1.16–1.39)^1^	<.01	<.01
Brain and NS (87,384)	1.31 (1.09–1.57)^1^	<.01	<.01	1.33 (1.06–1.66)^1^	.012	.018	1.28 (0.93–1.71)	.116	.116
Myeloma (85,862)	1.23 (1.09–1.37)^1^	<.01	<.01	1.17 (1.02–1.33)^1^	.025	.028	1.40 (1.13–1.72)^1^	<.01	<.02
Breast (826,612)	1.13 (1.09–1.17)^1^	<.01	<.01	0.90 (0.63–1.23)^1^	.923	.923	1.13 (1.09–1.17)^1^	<.01	<.01
Oral C&P (152,831)	1.12 (1.04–1.21)^1^	<.01	<.01	1.10 (1.01–1.20)^1^	.019	.025	1.24 (1.03–1.47)^1^	.017	.020
Prostate (953,458)	1.03 (1.01–1.05)^1^	<.01	<.01	1.03 (1.01–1.05)^1^	<.01	<.01	0.00 (0–0)	N/A	N/A

Abbreviations: CI, confidence interval; C&P, cavity and pharynx; NA, not applicable; NHL, non-Hodgkin lymphoma; NS, nervous system; SIR, standardized incidence ratio.

Adjusted *P*-values are as per Benjamini–Hochberg method for calculating false discovery rate.

1 Represent statistical significance, defined as *P* < .05.

Stratified by latency, most cancers (6 of 9) with an increased incidence of melanoma as a second cancer exhibited the highest risk of developing melanoma within 5 years of initial diagnosis. Melanoma (SIR = 17.6; 95% CI = 17.0–18.29), thyroid cancer (SIR = 1.80; 95% CI = 1.44–2.22), and oral cavity/pharynx cancer (SIR = 1.26; 95% CI = 1.02–1.54) were associated with the highest risk of developing a subsequent melanoma within 1 year of their original diagnoses. With prior leukemia (SIR = 1.78; 95% CI = 1.72–2.07), non-Hodgkin lymphoma (SIR = 1.49; 95% CI = 1.37–1.61), or breast malignancies (SIR = 1.21; 95% CI = 1.14–1.27), the risks of subsequent melanoma peaked within 1–5 years. The risks after myeloma (SIR = 1.44; 95% CI = 1.15–1.77) and prostate cancer (SIR = 1.04; 95% CI = 1.00–1.07) peaked 5–10 years after initial diagnoses. Brain and nervous system cancers had their highest risk 10–15 years after preliminary diagnosis (SIR = 2.0; 95% CI = 1.29–2.95). Notably, both melanoma and leukemia had statistically significant increased risks of subsequent melanoma throughout all the examined latency time frames (Table 2). After adjustment for false discovery rate, all unadjusted significantly different observations maintained their statistically significant differences (Table 1, Table 2).

**Table 2:** Risk of Melanoma Stratified by Latency and Age after Selected Primary Cancers

Primary Cancer	Latency Interval after Primary Cancer Diagnosis														
	2–11 mo			1–5 y			5–10 y			10–15 y			≥15 y		
	SIR	Unadjusted *P*-Value	Adjusted *P*-Value	SIR	Unadjusted *P*-Value	Adjusted *P*-Value	SIR	Unadjusted *P*-Value	Adjusted *P*-Value	SIR	Unadjusted *P*-Value	Adjusted *P*-Value	SIR	Unadjusted *P*-Value	Adjusted *P*-Value
Cutaneous melanoma	17.60^1^	<.01	<.01	10.80^1^	<.01	<.01	8.10^1^	<.01	<.01	7.05^1^	<.01	<.01	5.79^1^	<.01	<.01
95% CI	17.00–18.29^1^			10.53–11.03^1^			7.85–8.34^1^			6.76–7.36^1^			5.35–6.25^1^		
Leukemia	1.67^1^	<.01	<.01	1.89^1^	<.01	<.01	1.74^1^	<.01	<.01	1.46^1^	<.01	<.01	1.78^1^	<0.01	<0.01
95% CI	1.39–1.99^1^			1.72–2.07^1^			1.55–1.94^1^			1.21–1.75^1^			1.29–2.39^1^		
NHL	1.21^1^	.03	.04	1.49^1^	<.01	<.01	1.26^1^	<.01	<.01	1.21^1^	.01	.02	1.17	.23	.53
95% CI	1.02–1.42^1^			1.37–1.61^1^			1.14–1.39^1^			1.05–1.38^1^			0.90–1.50		
Thyroid	1.80^1^	<.01	<.01	1.33^1^	<.01	<.01	1.30^1^	<.01	<.01	1.16	.10	.19	1.24	.15	.45
95% CI	1.44–2.22^1^			1.17–1.49^1^			1.14–1.47^1^			0.97–1.38			0.92–1.64		
Brain and NS	0.89	.70	.70	1.48^1^	<.01	.01	1.17	.50	.50	2.00^1^	<.01	<.01	0.82	.86	.86
95% CI	0.54–1.39			1.11–1.95			0.76–1.73			1.29–2.95			0.22–2.69		
Myeloma	0.89	.53	.64	1.24^1^	.01	.01	1.44^1^	<.01	<.01	1.34	.17	.25	0.66	.63	.70
95% CI	0.63–1.23			1.05–1.45^1^			1.15–1.77^1^			0.87–1.96			0.14–1.93		
Breast	1.19^1^	<.01	<.01	1.21	<.01	<.01	1.10^1^	<.01	<.01	1.03	.49	.55	1.05	.44	.57
95% CI	1.07–1.32^1^			1.14–1.27^1^			1.03–1.16^1^			0.95–1.12			0.92–1.20		
Oral C&P	1.26^1^	.03	.04	1.17^1^	.01	.01	1.10	.19	.21	.92	.47	.55	1.18	.36	.54
95% CI	1.02–1.54^1^			1.04–1.31^1^			0.95–1.26^1^			0.73–1.14			0.82–1.65		
Prostate	1.02	.57	.64	1.03	.053	.053	1.04^1^	.03	.04	1.01	.58	.58	1.04	.30	.54
95% CI	0.95–1.1			1.00–1.07			1.00–1.07^3^			0.97–1.06			0.96–1.13		

**Primary Cancer**	**Age at Diagnosis of the Primary Cancer**														

	**<50 y**			**50–59 y**			**60–69 y**			**70–79 y**			**≥80 y**		
**SIR**	**Unadjusted *P*-Value**	**Adjusted *P*-Value**	**SIR**	**Unadjusted *P*-Value**	**Adjusted *P*-Value**	**SIR**	**Unadjusted *P*-Value**	**Adjusted *P*-Value**	**SIR**	**Unadjusted *P*-Value**	**Adjusted *P*-Value**	**SIR**	**Unadjusted *P*-Value**	**Adjusted *P*-Value**
Cutaneous melanoma	13.70^1^	<0.01	<.01	9.73^1^	<.01	<.01	8.85^1^	<.01	<.01	8.43^1^	<.01	<.01	9.40^1^	<.01	<.01
95% CI	13.20–14.15^1^			9.42–10.06^1^			8.59–9.11^1^			8.16–8.70^1^			8.98–9.84		
Leukemia	1.92^1^	<.01	<.01	1.84^1^	<.01	<.01	1.93^1^	<.01	<.01	1.52^1^	<.01	<.01	1.67^1^	<.01	<.01
95% CI	1.55–2.36^1^			1.59–2.11^1^			1.74–2.13^1^			1.35–1.71^1^			1.41–1.97^1^		
NHL	1.41^1^	<.01	<.01	1.33^1^	<.01	<.01	1.36^1^	<.01	<.01	1.30^1^	<.01	<.01	1.27^1^	<.01	<.01
95% CI	1.19–1.66^1^			1.18–1.49			1.24–1.49^1^			1.18–1.43^1^			1.09–1.47^1^		
Thyroid	1.37^1^	<.01	<.01	1.27^1^	<.01	<.01	1.22^1^	<.01	.01	1.34^1^	<.01	<.01	1.74^1^	<.01	<.01
95% CI	1.22–1.55^1^			1.11–1.46^1^			1.05–1.42^1^			1.09–1.62^3^			1.19–2.46^1^		
Brain and NS	1.87^1^	<.01	<.01	1.16	.50	.57	0.80	.39	.39	1.05	.96	1.0	1.66	.32	.36
95% CI	1.42–2.41^1^			0.76–1.69			0.47–1.26			0.57–1.76			0.61–3.62		
Myeloma	1.42	.19	.21	1.38^1^	.02	.02	1.39^1^	<.01	<.01	1.10	.40	.51	0.89	.57	.57
95% CI	0.83–2.28			1.05–1.79^1^			1.14–1.67^1^			0.88–1.36			0.60–1.26		
Breast	1.28^1^	<.01	<.01	1.05	0.17	.22	1.17^1^	<.01	<.01	1.05	.21	.31	1.15^1^	.01	.02
95% CI	1.19–1.38^1^			0.98–1.12			1.10–1.24^1^			0.97–1.13			1.03–1.28^1^		
Oral C&P	1.17	<.01	<.01	1.04	.63	.63	1.14^1^	.04	.05	1.00	1.0	1.0	1.61^1^	<.01	<.01
95% CI	0.90–1.48			0.89–1.21			1.00–1.29^1^			0.84–1.18			1.29–1.99^1^		
Prostate	1.21^1^	.04	.05	1.20^1^	<.01	<.01	1.04^1^	.01	.01	.97	.14	.25	.90	<.01	<.01
95% CI	1.01–1.45^1^			1.14–1.26^1^			1.01–1.07^1^			0.94–1.01			0.83–0.97		

Abbreviations: CI, confidence interval; C&P, cavity and pharynx; NHL, non-Hodgkin lymphoma; NS, nervous system; SIR, standardized incidence ratio.

Adjusted *P*-values are as per Benjamini–Hochberg method for calculating false discovery rate.

1 Represent statistical significance, defined as *P* < .05.

When comparing risk by age at initial cancer diagnosis, melanoma, leukemia, non-Hodgkin lymphoma, and thyroid cancers all had an increased risk of subsequent melanoma, regardless of age at primary diagnosis. Notably, subsequent melanoma risk increased 14-fold for those aged younger than 50 years with a prior melanoma (SIR = 13.7; 95% CI = 13.2–14.2). Other cancers that had an increased melanoma risk based on age of primary cancer onset included: myeloma between ages 50 and 70 years, brain and nervous system cancer in ages <50 years, breast cancer for all ages except 70–79 years, oral cavity/pharynx cancer for ages between 60 and 69 years and >80 years, and prostate cancer for ages <70 years (Table 2). We must note that it is difficult to identify biological reasons for the variations of the risks observed between the different age groups.

## Discussion

Overall, we identified 9 cancer types with increased risk of developing subsequent melanoma, each with sex, age, and latency-specific differences. The 3 primary cancers with the highest increased risks of subsequent melanoma were previous melanoma (865%), leukemia (76%), and non-Hodgkin lymphoma (33%). Increased surveillance may be a contributing factor to the increased risk we observed, particularly in patients with a prior melanoma. According to the American Academy of Dermatology clinical practice guidelines, patients with melanoma should undergo more frequent skin examinations, which can lead to detection bias (Swetter et al, 2019). However, increased screening alone may not adequately explain an 865% increased risk of melanoma development in patients with a prior melanoma. Another potential cause for the increased risk of melanoma after the cancers identified herein may be receipt of prior cancer therapies, particularly immunosuppressive regimens commonly used for lymphomas and leukemias (Kubica and Brewer, 2012).

Our analysis reveals certain at-risk groups, including those aged <50 years with a diagnosis of melanoma within the past year; individuals diagnosed with leukemia in their 60s; and lastly, those aged >80 years with thyroid cancer diagnosis within the past year. The at-risk groups defined in our study may benefit from a higher degree of clinical suspicion for melanoma when being evaluated by physicians. In addition, obtaining a detailed oncological history pertaining to the 9 cancer types with increased risk of subsequent melanoma may help dermatologists in risk stratifying patients for skin cancer screenings.

The limitations of this study include potential ascertainment bias and patient migration in and out of areas covered by Surveillance, Epidemiology, and End Results registries. These findings solely reflect the data available in the Surveillance, Epidemiology, and End Results cancer registries. Additional large-scale population studies are needed to confirm these findings and to investigate interventions aimed at improving outcomes in these patients.

## Materials and Methods

Each primary malignancy was queried utilizing its associated Site Recode ICD-O-3/World Health Organization 2008, and patients were excluded if the report was obtained solely from a death certificate or autopsy report with no confirmation of diagnosis. Similarly, patients who received a diagnosis of cutaneous melanoma within 2 months of their primary cancer diagnosis were excluded owing to the high likelihood of these being the same primary process rather than 2 independent malignancies. The date of the first primary diagnosis was used as the initial date from which outcome latencies were calculated. Relative risk was calculated using SIR, defined as the ratio between observed and expected cases (SIR = observed/expected). Statistical significance was established on the basis of 95% CI. To adjust for multiple comparisons, we utilized the Benjamini–Hochberg method for calculating false discovery rates (Benjamini and Hochberg, 1995). Using this method, the unadjusted and adjusted *P*-values across multiple primary cancers were calculated.

## Ethics Statement

This study utilizing a Surveillance, Epidemiology, and End Results–generated cohort was exempt from institutional review board approval.

## Data Availability Statement

The data underlying this article are publicly available in both the article itself and the Surveillance, Epidemiology, and End Results database (https://seer.cancer.gov/data-software/).

## ORCIDs

Thomas Z. Rohan: http://orcid.org/0009-0005-5378-9695

Jenna L. Mandel: http://orcid.org/0000-0001-9994-6469

Henry Y. Yang: http://orcid.org/0009-0002-5266-6878

Lauren Banner: http://orcid.org/0009-0006-2517-7787

Daniel Joffe: http://orcid.org/0000-0001-6708-960X

Rachel Zachian: http://orcid.org/0009-0002-4502-7517

Jaanvi Mehta: http://orcid.org/0000-0002-5181-1888

Safiyyah Bhatti: http://orcid.org/0009-0000-0267-1607

Tingting Zhan: http://orcid.org/0000-0001-9971-4844

Neda Nikbakht: http://orcid.org/0000-0002-9587-8689

## Conflict of Interest

NN serves on the advisory board for Helsinn Therapeutics and Kyowa Hakko Kirin. The remaining authors state no conflict of interest.

## References

[bib1] American Cancer Society (2023). Cancer facts and statistics. http://cancerstatisticscenter.cancer.org.

[bib2] Benjamini Y., Hochberg Y. (1995). Controlling the false discovery rate: a practical and powerful approach to multiple testing. J R Stat Soc Series B Stat Methodol.

[bib3] Demoor-Goldschmidt C., de Vathaire F. (2019). Review of risk factors of secondary cancers among cancer survivors. Br J Radiol.

[bib4] Johansson M., Brodersen J., Gøtzsche P.C., Jørgensen K.J. (2019). Screening for reducing morbidity and mortality in malignant melanoma. Cochrane Database Syst Rev.

[bib5] Kubica A.W., Brewer J.D. (2012). Melanoma in immunosuppressed patients. Mayo Clin Proc.

[bib6] Morton L.M., Onel K., Curtis R.E., Hungate E.A., Armstrong G.T. (2014). The rising incidence of second cancers: patterns of occurrence and identification of risk factors for children and adults. Am Soc Clin Oncol Educ Book.

[bib7] Siegel R.L., Miller K.D., Wagle N.S., Jemal A. (2023). Cancer statistics, 2023. CA Cancer J Clin.

[bib8] Swetter S.M., Tsao H., Bichakjian C.K., Curiel-Lewandrowski C., Elder D.E., Gershenwald J.E. (2019). Guidelines of care for the management of primary cutaneous melanoma. J Am Acad Dermatol.

[bib9] Ugai T., Sasamoto N., Lee H.Y., Ando M., Song M., Tamimi R.M. (2022). Is early-onset cancer an emerging global epidemic? Current evidence and future implications. Nat Rev Clin Oncol.

